# Supplementary material for: Relationship between stress hyperglycemia ratio of one-year mortality in patients with heart failure: Analysis of the MIMIC-IV database
Source: PLoS One. 2025 Aug 8;20(8):e0328812. doi: 10.1371/journal.pone.0328812 (PMC12333993; doi:10.1371/journal.pone.0328812)
Supplement: S1 File — International Classification of Diseases (ICD) code. (DOCX) [file pone.0328812.s001.docx]

**Supplementary material**

Table S1: ICD code

| Variable | Code |  |
| --- | --- | --- |
| Acute CHF | I5021 | ICD10 |
| Chronic CHF | I5022 |  |
| Acute on chronic CHF | I5023 |  |
| Diastolic HF | I503 |  |
| Unspecified DHF | I5030 |  |
| Acute DHF | I5031 |  |
| Chronic DHF | I5032 |  |
| Acute on chronic DHF | I5033 |  |
| Combined CHF, DHF | I504 |  |
| Unspecified combined | I5040 |  |
| Acute combined | I5041 |  |
| Chronic combined | I5042 |  |
| Acute on chronic combined | I5043 |  |
| Left HF | 4281 | ICD9 |
| SHF (unspecified) | 42820 |  |
| ASHF | 42821 |  |
| CSHF | 42822 |  |
| Acute on Chronic SHF | 42823 |  |
| DHF (unspecified) | 42830 |  |
| ADHF | 42831 |  |
| CDHF | 42832 |  |
| Acute on chronic DHF | 42833 |  |
| Combined (unspecified) | 42840 |  |
| Acute combine | 42841 |  |
| Chronic combine | 42842 |  |
| Acute on chronic combine | 42843 |  |
| HF (unspecified) | 4289 |  |
| Primary hypertension | I10 | ICD10 |
| Malignant essential hypertension | 4010 | ICD9 |
| Benign hypertension | 4011 |  |
| Unspecified hypertension | 4019 |  |
| Secondary DM (diabetes mellitus) without complication | 24900,24901, | ICD9 |
| DM without complication, type II or unspecified | 25000,25002 |  |
| T1DM no complication | 25001,25003 |  |
| T1DM | E10 | ICD10 |
| T1DM without complication | E109 |  |
| T2DM | E11 | ICD10 |
| T2DM without complication | E119 |  |
| Other specified DM without complications | E139 |  |
| Angina decubitus | 4130 | Icd9 |
| Prinzmetal angina | 4131 |  |
| Unspecified angina pectoris | 4139 |  |
| Angina pectoris | I20 | Icd10 |
| Unstable angina | I200 |  |
| Other angina | I201, I208, I209 |  |
| Acute myocardial infarction (MI) | 41000，41001，41002，41010，41011，41012，41020，41021，41022，41030，41031，41032，41040，41041，41042，41050，41051，41080，41081，41082，41090，41091，41092， | Icd9 |
| Acute myocardial infarction | I21 | Icd10 |
| STEMI | I210, I2101, I2109, I211, I2111, I2119, I212, I2121, I2129, I213 |  |
| NSTEMI | I214 |  |
| Unspecified AMI | I219 |  |
| Other type AMI | I21A |  |
| MI type 2 | I21A1 |  |
| Old AMI | 412 | Icd9 |
| Old MI | I252 | Icd10 |
| Coronary atherosclerosis of native coronary artery | 41401 | 9 |
| Coronary atherosclerosis due to calcified coronary lesion | 4144 |  |
| Coronary atherosclerosis due to calcified coronary lesion | I2584 | Icd10 |
| AF (atrial fibrillation) | 42731 | 9 |
| AF and AFL (atrial flutter) | I48 | 10 |
| PAF | I480 |  |
| Per AF | I481 |  |
| Longstanding persistent AF | I4811 |  |
| Other per AF | I4819 |  |
| Chronic AF | I482 |  |
| Chronic AF, unspecified | I4820 |  |
| Permanent AF | I4821 |  |
| Unspecified AF/AFL | I489 |  |
| Unspecified AF | I4891 |  |
| CKD (chronic kidney disease) I | 5851 | Icd9 |
| CKD II | 5852 |  |
| CKD III | 5853 |  |
| CKD IV | 5854 |  |
| CKD V | 5855 |  |
| CKD unspecified | 5859 |  |
| CKD | N18 | Icd10 |
| CKD I | N181 |  |
| CKD II | N182 |  |
| CKD III | N183 |  |
| CKD IV | N184 |  |
| CKD V | N185 |  |
| CKD unspecified | N189 |  |

Figure S1: Flow chart of population.

Figure S2 One-year mortality Kaplan-Meier curves in participants a) with AHF and b) with CHF

AHF: acute heart failure; CHF: chronic heart failure

Figure S3 Restricted cubic spline (RCS) in participants a) with AHF and b) with CHF

AHF: acute heart failure; CHF: chronic heart failure

Table S1 Sensitivity analysis conducted among patients scored with systemic inflammatory response syndrome (SIRS) and the simplified acute physiology score (SAPS) II

|  | HR | 95%CI |
| --- | --- | --- |
| SHR (categorical) |  |  |
| Model 1 | 1.325 | 1.095-1.603 |
| Model 2 | 1.021 | 1.017-1.025 |

Model 1, adjust age, gender, race, SIRS, history of ACS, HT, ACEI/ARB, digoxin, dopamine/dobutamine, thiazine diuretic, RBC, WBC, PLT and hemoglobin.

Model 1, adjust age, gender, race, SAPS II, history of ACS, HT, ACEI/ARB, digoxin, dopamine/dobutamine, thiazine diuretic, RBC, WBC, PLT and hemoglobin.
