# Supplementary figures and images for: Relationship between stress hyperglycemia ratio of one-year mortality in patients with heart failure: Analysis of the MIMIC-IV database
Source: PLoS One. 2025 Aug 8;20(8):e0328812. doi: 10.1371/journal.pone.0328812 (PMC12333993; doi:10.1371/journal.pone.0328812)

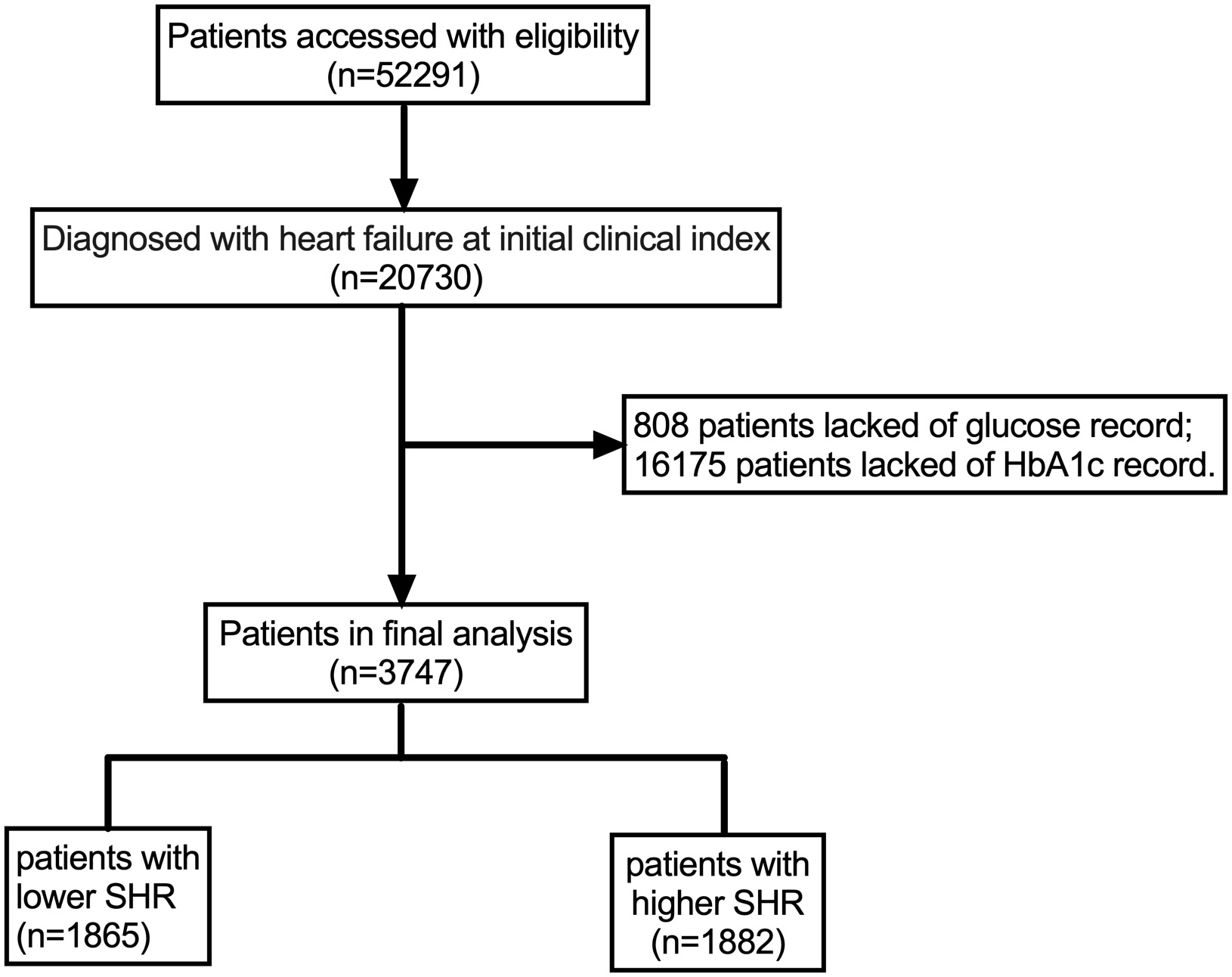

Supplement: S1 Fig — (JPG) [file pone.0328812.s002.jpg]

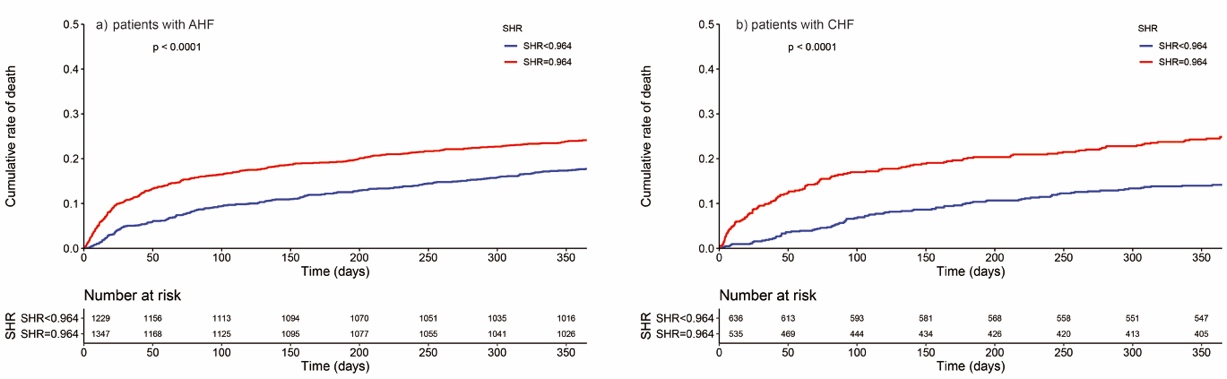

Supplement: S2 Fig — (JPG) [file pone.0328812.s003.jpg]

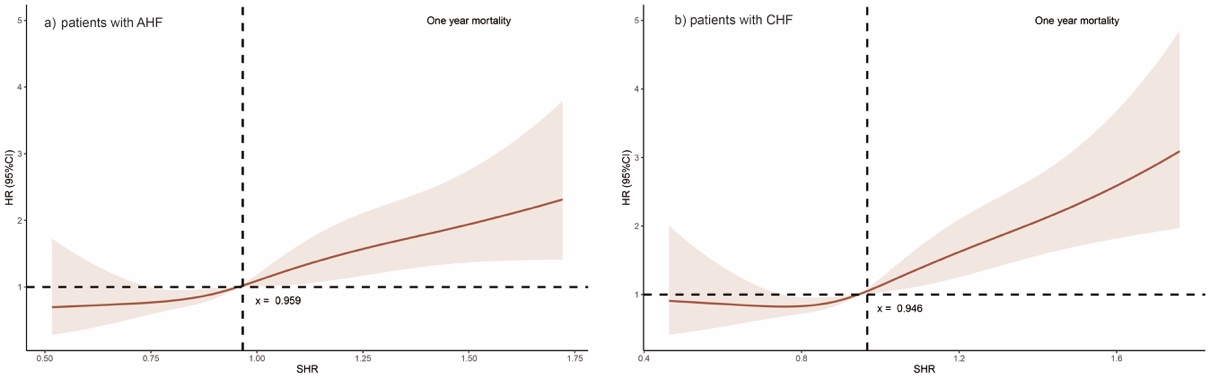

Supplement: S3 Fig — (JPG) [file pone.0328812.s004.jpg]
