# Supplementary material for: Relationship between stress hyperglycemia ratio of one-year mortality in patients with heart failure: Analysis of the MIMIC-IV database
Source: PLoS One. 2025 Aug 8;20(8):e0328812. doi: 10.1371/journal.pone.0328812 (PMC12333993; doi:10.1371/journal.pone.0328812)
Supplement: Attachment — Submitted filename: Point to point2.docx [file pone.0328812.s007.docx]

**Point-to-point response**

Dear Editors and Reviewers,

Thank you very much for providing us with the opportunity to revise our manuscript.

We are grateful to the editor and reviewers for their constructive comments and helpful suggestions. Responding to the critics, we have conducted additional analyses as requested. We also improved our expression in background and discussion. We believe that our manuscript has been substantially improved and hope it is now acceptable for the Thrombosis Journal.

I assure you that all authors have read and approved the submission of the revised manuscript. Also, this work containing the original research has not been under consideration for publication elsewhere.

Please let me know if you have any further questions.

With warmest regards,

Yanmin Yang,

Emergency and Critical Care Center, Fuwai Hospital, National Center for Cardiovascular Diseases of China, Chinese Academy of Medical Sciences and Peking Union Medical College, Beijing, China

E-mail: [yyminfuwai@163.com](mailto:yyminfuwai@163.com)

The main corrections in the paper and the responses to the reviewer’s comments are as follows (All the text in bold and underlined font are the original comments from the editor/reviewers and our response is in normal font):

**To reviewer 1:**

**This study focuses on the association between metabolic stress, insulin resistance, and heart failure prognosis. From the perspective of metabolic abnormalities in heart failure, the following studies may provide further insights into the significance of SHR: ESC Heart Fail. 2023;10:32-43. Heart Vessels. 2021;36:965-977. J Cardiol. 2020;75:689-696.**

Thank you for your insightful comments. We have added the association between metabolic conditions and heart failure prognosis in the introduction section and have cited the references as per your recommendation. (page 3,line 45-46)

**Although the somewhat limited AUC of 0.56 in the ROC analysis is briefly mentioned, I feel that a more in-depth discussion on its clinical applicability and practical significance would be beneficial.**

Thanks for your constructive suggestions. We added a discussion on its clinical applicability and practical significance in discussion section.(Page 14, line 270-275)

**To reviewer 2:**

**Special thanks for reviewer 2.**

**To reviewer 3:**

**Special thanks for reviewer 3.**

**To reviewer 4:**

**Special thanks for reviewer 4.**
